# Feature Identification of Compensatory Gene Pairs without Sequence Homology in Yeast

**DOI:** 10.1155/2012/653174

**Published:** 2012-08-16

**Authors:** Chien-Hua Peng, Shu-Hsi Lin, Shih-Chi Peng, Ping-Chiang Lyu, Masanori Arita, Chuan-Yi Tang

**Affiliations:** ^1^Department of Resource Center for Clinical Research, Chang Gung Memorial Hospital, Kueishan, Taoyuan 333, Taiwan; ^2^Institute of Bioinformatics and Structural Biology, National Tsing Hua University, Hsinchu 300, Taiwan; ^3^Department of Nuclear Medicine and Molecular Imaging Center, Chang Gung Memorial Hospital and Chang Gung University, Taoyuan 333, Taiwan; ^4^Department of Medical Science, National Tsing Hua University, Hsinchu 300, Taiwan; ^5^Department of Biophysics and Biochemistry, The University of Tokyo, Tokyo 113-0033, Japan; ^6^Metabolomics Research Division, RIKEN Plant Science Center, Yokohama, Kanagawa 230-0045, Japan; ^7^Department of Computer Science, National Tsing Hua University, Hsinchu 300, Taiwan; ^8^Department of Computer Science and Information Engineering, Providence University, Taichung 200, Taiwan

## Abstract

Genetic robustness refers to a compensatory mechanism for buffering deleterious mutations or environmental variations. Gene duplication has been shown to provide such functional backups. However, the overall contribution of duplication-based buffering for genetic robustness is rather small. In this study, we investigated whether transcriptional compensation also exists among genes that share similar functions without sequence homology. A set of nonhomologous synthetic-lethal gene pairs was assessed by using a coexpression network, protein-protein interactions, and other types of genetic interactions in yeast. Our results are notably different from those of previous studies on buffering paralogs. The low expression similarity and the conditional coexpression alone do not play roles in identifying the functionally compensatory genes. Additional properties such as synthetic-lethal interaction, the ratio of shared common interacting partners, and the degree of coregulation were, at least in part, necessary to extract functional compensatory genes. Our network-based approach is applicable to select several well-documented cases of compensatory gene pairs and a set of new pairs. The results suggest that transcriptional reprogramming plays a limited role in functional compensation among nonhomologous genes. Our study aids in understanding the mechanism and features of functional compensation more in detail.

## 1. Introduction

Genetic robustness is critical for enhancing organism's capability to tolerate random mutations [1]. One of the features for biological robustness is functional redundancy, in which two or more components can perform similar functions [2]. From a theoretical perspective, two main mechanisms have been proposed for explaining biological robustness due to functional redundancy [3]. The first mechanism is duplicate buffering [4, 5], which is a backup compensation for the loss or mutation of a duplicate (paralog), to overcome stochastic fluctuations in gene and protein expression [3–6]. This has been considered as an obvious source of genetic redundancy that can compensate for a gene loss [4, 5, 7, 8]. More specifically, if gene A and gene B are functionally redundant duplicates, the expression of gene B will be upregulated to rescue the organism upon the mutation of gene A [6]. However, functionally redundant duplicates are evolutionarily unfavorable [9]. The capability to compensate for gene mutations may be lost over long periods because of divergence [9–14]. 

The second mechanism stems from the viewpoint of distributed robustness, usually achieved through degeneracy. Degeneracy refers to a circumstance where structurally distinct components bear out similar or partially overlapping functions [2, 3, 8, 15]. Especially, alternative pathways are found to provide the robustness of the metabolic network [16], regulatory network [17, 18], and signal transduction network [19]. Such systems-level redundancy is not mediated by duplicates but by evolutionarily distant proteins. Degeneracy may contribute more to the overall robustness than duplicate buffering [20]. 

In prokaryotes and eukaryotes, a high proportion of gene mutations do not affect phenotypically [4, 8, 21–25]. Double knockout of both duplicate genes shows a significantly larger defect on phenotypes than expected from the effects of single knockouts [4, 26], but previous analyses found the contribution of duplicates to genetic buffering is only around 23% or even less [4, 27, 28]. As Ihmels et al. suggested, homologous duplication may not be a prerequisite for gene backup capability [27]. On the other hand, Kafri et al. proposed that transcriptional reprogramming is the major factor of functional compensation: when one gene is mutated, the expression of another is reprogrammed to recover the original function [6]. 

The transcriptional reprogramming model suggested by Kafri et al. can be extended to explain the compensatory phenomenon caused by distributed robustness. In this study, we focused on nonhomologous genes and identified potential gene pairs compensated by distributed robustness. One conceivable indicator for functional compensation is the synthetic- or sick-lethal (SSL) relationship. SSL interactions occur between two genes whose disruptions in combination yield a stronger growth defect than that generated by either single disruption. SSL pair is likely to be functionally equivalent or share partially overlapping functions. Although transcriptional compensation between SSL gene pairs seems to be rare and play a limited role in maintaining robustness [29, 30], the large-scale techniques provide abundant experimental data for the study of genetic compensation on a genome-wide level. 

In order to determine whether transcriptional compensation occurs among nonhomologous genes, we extracted 6186 nonhomologous SSL interactions from BioGRID Interaction Database [31]. Of these SSL gene pairs, only 171 pairs were found to have the potential compensatory capability. We provide evidences that the degree of sharing regulatory elements between the SSL gene pairs and the ratio of common neighbors in the biological network are related to compensation capability. Moreover, most of the nonhomologous compensatory genes are multifunctional interaction hubs. The overall effect of robustness contributed from functional redundancy is still an issue of debate. Transcriptional reprogramming is not the only mechanism to achieve the functional compensation among nonhomologous genes, but our analysis would provide a unique viewpoint of genetic robustness beyond duplication-based compensation. 

## 2. Materials and Methods

### 2.1. Dataset of Synthetic Lethal Genes and mRNA Expression Data

With systematic generation of double mutant strains of *Saccharomyces cerevisiae* [32–34], two approaches, synthetic genetic arrays (SGA) and diploid-based synthetic lethality analysis on microarrays (dSLAM), have been developed to identify genome-wide synthetic lethal interactions. We collected all experimentally verified SSL interactions from BioGrid database (http://www.thebiogrid.org/). Recent data sets of high-density epistatic miniarray profiles (E-MAPs) were also included [35, 36]. Temporal mRNA expression data for 6359 *S. cerevisiae* genes in 40 natural and perturbed conditions were obtained from ExpressDB [37]. Genome-wide responses to 259 single-gene mutants were also collected [38]. All expression profiles of the genes in each condition were standardized with respect to the mean and variance.

### 2.2. Calculation of Sequence Similarity and Assignment of Functional Module

For each pair of synthetic lethal genes, the corresponding protein sequences were downloaded from NCBI RefSeq (release 24). We defined nonhomologous protein pairs as two sequences that, by BLASTP with standard parameters, share less than 30% identity. Of 9237 SSL pairs, 6186 were nonhomologous. Tong et al. reported that 98% of SSL gene pairs were nonhomologous [34], but our criterion was stricter: only 67% of SSL pairs were retained for further functional analyses.

Functional modules applied on our dataset were collected from the work by Petti and Church [39]. They defined 72 functional modules from the *S. cerevisiae *genome database at MIPS (Munich Information Center for Protein Sequences) [40]. 

### 2.3. Biological Network Construction

We constructed two types of biological networks, the molecular functional network and the gene coexpression network, to estimate the functional similarity between two genes.

The 40 expression profiles under natural and perturbed conditions were used to construct the coexpression network. To evaluate the degree of coexpression between each pair of genes, Pearson correlation coefficients (PCCs) under different conditions were calculated and genes with PCC > 0.7 (upper 5th percentiles) were connected.

As for the functional network, we collected protein-protein and genetic interaction data from the BioGRID (http://www.thebiogrid.org/) and BOND (http://bond.unleashedinformatics.com/) databases.

### 2.4. Promoter Regulatory Elements Analyses

Promoter sequences of all nonhomologous SSL genes with functional similarity were retrieved from NCBI RefSeq (release 24) (http://www.ncbi.nlm.nih.gov/RefSeq/). A set of 103 yeast regulatory elements (motifs) and their gene assignments were collected from the TRANSFAC database (version 11.3) (http://www.gene-regulation.com/pub/databases.html), which records all experimentally verified transcription factors of *S. cerevisiae* and their target genes.

For each pair of nonhomologous SSL genes with functional similarity, a pattern search program in TRANSFAC (pMatch) was applied to match both promoter sequences individually with all experimentally determined regulatory motifs. Then, the motif-content overlap (MCO) score [6] was calculated as follows:

(1) $$\begin{array}{c} \text{MCO}=\frac{\left|r_{1}\cap r_{2}\right|}{\max\left(\left|r_{1}\right|,\left|r_{2}\right|\right)}, \end{array}$$

where *r*_1_ and *r*_2_ are sets of matched regulatory elements in promoter one and promoter two, respectively. |*r*_*x*_| denotes the number of regulatory elements in gene *x*.

### 2.5. MES and PCoR Analyses

MES and PCoR defined by Kafri et al. stand for “mean expression similarity” and “partial coregulation”, respectively [6]. For each pair of the nonhomologous SSL pairs with high functional similarity, Pearson correlation coefficients of mRNA expression profiles under 40 different conditions were calculated to compute these scores.

### 2.6. Functional Relatedness Analyses

For all gene pairs, we defined the CN (common neighbor) score as a measure for the fraction of shared partners. The score was defined as

(2) $$\begin{array}{c} \text{CN}=\frac{n_{12}}{\left(n_{1}+n_{2}-n_{12}\right)}, \end{array}$$

where *n*_1_ and *n*_2_ denote the number of neighbors for one and the other synthetic lethal counterpart, respectively, and *n*_12_ denotes the number of common neighbors shared between the two SSL genes. If the two SSL genes are connected directly, the value of *n*_12_ is two. The significance of the CN score was estimated by a *P* value, (Pr(CN_random_≧CN)), computed by randomly sampling sets of 10^5^ pairs of genes. 

## 3. Results

### 3.1. Sequence Similarity and Functional Module Analysis

 The central issue is whether transcriptional compensation plays a significant role for nonhomologous genes with similar functions. To exclude all homologous SSL gene pairs, we first evaluated a set of SSL pairs of *S. cerevisiae* through sequence similarity. Of 9237 SSL pairs, 6186 were nonhomologous and retained for further analyses. Next, we identified 1771 nonhomologous SSL pairs that are categorized into either one of 72 functional modules [39], which are defined as a group of genes or proteins involved in a common cellular process in the gene ontology. 

### 3.2. Gene Expression Analyses

**Figure 1:**
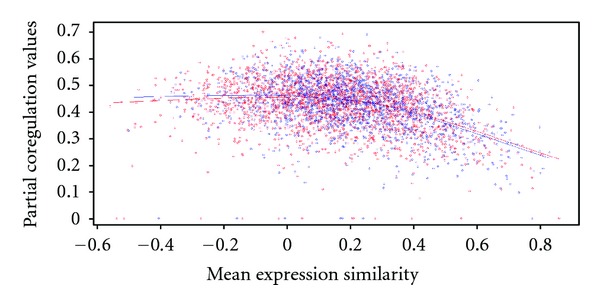
The distribution of MES and PCoR for synthetic sick/lethal (SSL) genes in the same and different functional modules. SSL genes in the same functional modules (blue) and different functional modules (red) are plotted as functions of the mean expression similarity (MES) and partial coregulation values (PCoR). The trends of both dataset are very similar. A two-sample *t*-test reveals that the differences between them are statistically nonsignificant as LOWESS (locally weighted scatterplot smoothing) curves show. Both *P* values for MES and PCoR are around 0.19.

Kafri et al. showed that the compensatory capability of a gene pair is optimal when its mean expression similarity (MES) falls from 0 to ~0.2 and the standard deviation of gene expression correlations (PCoR) higher than 0.4 [6]. To examine whether this feature also exists in the selected 1771 gene pairs, we plotted their scores on a plane spanned by MES and PCoR (blue circles in Figure 1) against the remaining nonhomologous SSL pairs that were not mapped into the same functional modules (red circles in Figure 1). The two-sample *t*-test revealed that the difference between the two sets was not statistically significant (*P* values are 0.59 and 0.91 for MES and PCoR, resp.). We also analyzed the distribution of both PCoR and MES for homologous SSL genes. In this case, the *P* values of the two-sample *t*-test for MES and PCoR were 0.000038304 and 0.000176, respectively. Our results demonstrated that the measurements of PCoR and MES did not fully delineate compensatory capability in nonhomologous genes. 

### 3.3. Network-Based Modeling

Paralogs with backup capacity have high propensity to be coclustered in the same protein complexes and share common interacting partners [41]. To further filter nonhomologous pairs with high functional similarity, we defined the CN (common neighbor) score (see Section 2). 

We used two complementary biological networks: the molecular functional network and the gene coexpression network. Both types of networks were constructed to estimate the functional similarity between two genes (see Section 2).

**Figure 2:**
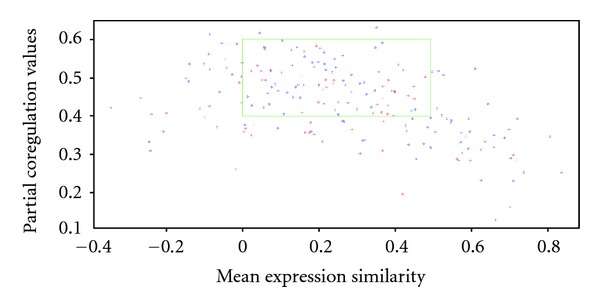
The distribution and characterization of the PCoR and MES for the 171 nonhomologous genes with a significant CN score and the genes with potential compensatory capability. 171 nonhomologous SSL genes with a significant CN score are plotted in blue color. The selected genes with potential compensatory capability from the gene expression profiles are plotted in red. Seventy percent of the 171 genes (in a green rectangle) have the values of MES and PCoR concentrated in [0, 0.5] and [0.4, 0.6], respectively. The selected genes are also concentrated in the aforementioned range (in a green rectangle).

Of the 1771 nonhomologous SSL pairs in the same functional module, 171 pairs had significant CN scores (*P*≦0.01) in both the molecular functional network and the coexpression network.  Figure 2 shows the distribution of these 171 SSL pairs on the MES-PCoR plane. About 70% of these functionally overlapping nonhomologous SSL pairs had MES values between 0 and 0.5 as well as PCoR values from 0.4 to 0.6. The above MES and PCoR values suggest that expression patterns of nonhomologous SSL pairs are conditionally coexpressed. The distribution of MES and PCoR values for the 171 nonhomologous pairs showed a trend similar to that observed for paralogous backup genes [6]. Based on the gene expression profiles corresponding to diverse mutations [38], we selected a set of genes as potential compensatory genes, which show expression alteration after mutation or deletion of their SSL partner.  Figure 2 illustrates the distribution of PCoR and MES for the 171 nonhomologous genes (in blue color) and selected potential compensatory genes from (in red color). The values of the PCoR and MES are more clustered in the intervals [0.4, 0.6] and [0, 0.5], respectively. To further investigate the distribution of PCoR and MES, both measurements were also estimated on CN-significant and non-CN-significant nonhomologous genes in the same functional module. In this case, PCoR and MES were not associated with the CN score (*P* values of 2-sample *t*-test are 0.07 and 0.97 for MES and PCoR, resp.). On the other hand, for homologous SSL pairs in the same functional module, MES and PCoR are statistically significant between CN-significant and non-CN-significant pairs (*P* values of 2-sample *t*-test are 0.002279 and 0.0197 for MES and PCoR, resp.). Again, these two measurements failed to find cofunctional compensatory pairs when they are not homologous. 

### 3.4. Motif-Sharing Analysis of Nonhomologous SSL Pairs

Because the maximal duplicate-associated compensatory capability might coincide with intermediate levels of motif sharing in the promoter regions of the backup gene pair, partial similarity of regulatory controls may form the basis of transcriptional reprogramming in response to the loss of one paralogous partner [6]. Hence, we next also investigated the regulatory elements in the promoter sequences of the functionally overlapping nonhomologous SSL pairs and calculated the MCO score for each pair. 

**Figure 3:**
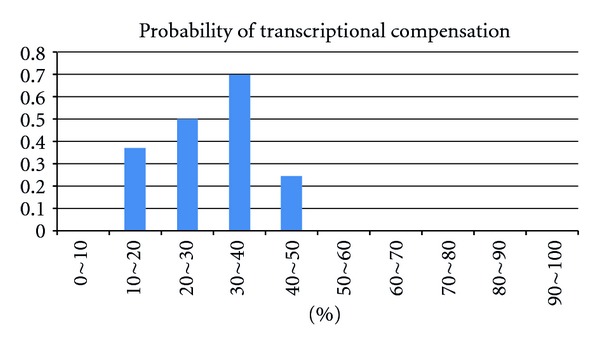
The probability of transcriptional compensation and the score of the motif-content overlap. In this analysis, SSL pairs were considered to have functional compensation potential only if they were in the same functional module and had significant ratios of common neighbors. Furthermore, the logarithm of the transcriptional response to deletion of the counterpart member was at least 1.5 (*P* ≤ 0.01). The number of qualified compensation—providing candidate pairs (log ratio ≥1.5) was divided by the total number of functionally characterized pairs (regardless of the log ratio) for each of the ranges of motif-overlapped ratios.

We used the aforementioned mRNA expression profiles of single-gene mutants to study the relationship between sharing regulatory elements and the compensation capability of nonhomologous SSL pairs. Of the 259 knockouts in this expression dataset, 66 nonhomologous genes with functional similarity had synthetic lethal interactions.  Figure 3 presents the relationship between MCO scores and the corresponding probabilities of functional compensation. The greatest proportion of compensatory genes was observed to have MCO scores between 30% and 40%. Similar to compensation by duplicate genes, backup capability was obtained when two genes share only part of their regulatory elements. It should be noted that highly similar regulatory controls in a gene pair result in strongly correlated expression profiles. In this scenario, both genes might be expressed at similar levels and might be expected to be required simultaneously. This may be why nonhomologous SSL pairs with overlapping functions lack the potential for compensation capability when their MCO score is higher than 0.5. Although the analysis of the mRNA expression data is not comprehensive, this analysis provides an evidence to show that partial overlap of the sharing regulatory element is one of the features of compensatory genes. 

Taken together, we proposed that the aforementioned ratio of common neighbors in the biological network and the score of the motif-content overlap are critical determinants of the compensatory capability for nonhomologous genes.

### 3.5. Identification of Functionally Compensatory Genes

As described above, three features were investigated for nonhomologous compensatory genes: (i) synthetic lethality; (ii) the ratio of common neighbors in the biological network; (iii) partial overlap of the regulatory elements of two genes. With these parameters, we selected nonhomologous functionally compensatory genes (see the supplementary table). 89.3% of the selected compensation pairs were over-expressed (log_2_ fold change > 3) when its partner was mutated [38] (see the supplementary table in Supplementary Material available online at doi:10.1155/2012/653174). Consistent with the previous study [30], transcriptional compensation between SSL gene pairs may only appear in a small portion. Many of them were associated with signal transduction, metabolic processes, ribosomal proteins, and posttranslational protein modification (see the supplementary table for the detail function of each gene). The compensation mechanisms between genes seemed to be highly divergent [42]. We took two candidate pairs, *kar2*/*sil1 *and* kar2*/*lhs1*, as examples. The former, *kar2* and *sil1, *shares common regulatory elements in their promoters [43] and exhibits synthetic lethal phenotype [44]. Similarly,* lhs1* and *sil1* also exhibit SSL interaction [45]. Both SIL1 and LHS1 are nucleotide exchange factors of KAR2 and were proposed to bind KAR2 in a mutually exclusive manner [46]. The promoter of *lhs1* was observed to be transcriptionally induced when the mutation occurs in *kar2* [47, 48]. On the other hand, at least in the function for the protein translocation, overexpressed SIL1 can partially compensate for LHS1 during loss of *lhs1*. However, this compensation seems not originating from modulation through *kar2* [45]. The mechanism of robustness among these three genes therefore cannot be simply explained by transcriptional reprogramming. Another example of a nonhomologous compensatory gene pair is provided by two members of the RAD2 nuclease family, *rad27* and *exo1*. Functional overlap of RAD27 and EXO1 was observed from identical structure-specific endonuclease and 5′ exonuclease activities. The overexpression of EXO1 results in the suppression of multiple *rad27* null mutation-associated phenotypes. Interestingly, similar compensatory behaviors were found in RAD2, but in complementing a different type of mutation, that is, base excision repair. EXO1 and RAD2 complement the defects of the *rad27* mutant to different extents. These results suggest that compensation of RAD27 can be achieved in an alternative way [49]. Other examples, such as the chitin synthase gene *chs3* and the *β*-1, 3-glucan synthase gene* fks1*: mutations in fks1 results in upregulation of CHS3 [29, 50]. As the above examples suggest, the compensation mechanism of nonhomologous genes might occur beyond the level of transcriptional reprogramming.

## 4. Discussion

Kafri et al. defined MES and PCoR measurements to estimate mRNA expression patterns across different conditions for each pair of paralogs [6]. Backup behaviors were rarely found in similarly expressed paralogs [3, 27]. However, some gene pairs with differentially regulated profiles compensated for each other's loss. This compensation was proposed to be involved with responsive backup circuits rather than through direct functional compensation [51]. It was also found that PCoR, which represents the switching capability between similar and dissimilar expression profiles, was a strong predictor of paralogous backup gene pairs [6]. 

However, in the complete set of 10,819 SSLs, Stein and Aloy found that only 2.5% are gene duplicates, whereas 35.7% are pathway redundancy genes [52]. Analyses of compensatory pairs revealed that roughly 20–35% of the compensations were due to paralogous genes, and a similar result was also observed in the previous report [27]. The overall contribution of paralogous genes to genetic robustness was found to be overestimated [28]. Therefore, we focused on nonhomologous genes and used a network-based approach to identify putative nonhomologous functionally compensatory gene pairs. We first assessed the sequence similarity of SSL pairs and identified nonhomologous genes with partially overlapping function as potential compensatory genes. Unlike the prediction for paralogous compensatory genes, our results showed that the MES and PCoR alone are insufficient to identify nonhomologous compensatory genes. The mechanism of functional compensation of nonhomologous genes may be more complicated and different from duplication-based compensation. 

The degree of functional relatedness seems to be an important feature of compensatory genes. For each pair of genes, the ratio of common neighbors in the molecular functional and coexpression networks appears to be a reliable measurement for functional relatedness. Furthermore, as duplicate-associated backup genes, most of the multifunctional backup genes only compensate one of their partner's functions [27]. This suggests that compensatory interactions are intricate and context-sensitive. A recent study has showed that paralog responsiveness for deletion of their duplicate genes is environmental requirement [53]. Consistent with previous reports, our results also showed that most compensatory pairs do not share promoter motifs (see Figure 3). Therefore, it is reasonable to suppose that partially sharing motifs might allow organisms to adapt for upregulation by common regulatory factors and also to provide compensation under certain conditions. However, this transcriptional backup is only one of possible mechanisms for genetic functional compensation.

In a recent report, a method for inferring genetic networks, the stepwise structural equation modeling algorithm (SSEM), was developed for prediction of transcriptional compensation interactions [54]. This model incorporated structural equation modeling and various model selection criteria to infer compensation interactions for small groups of genes that are synthetic sick or lethal. SSEM uses time course expression patterns to predict compensatory gene pairs. However, SSL gene pairs with similar or compensatory expression patterns do not always share the same function. To overcome this problem, our approach incorporated the functional relatedness of two compensatory gene candidates using a network-based method and covered SSL interaction data at the whole-genome scale. In particular, assessment of physical and genetic interactions for each compensatory candidate increased the confidence that the resulting compensatory pairs are functionally associated. When no abundant time course data are available, the compensatory gene pairs can be identified by applying our framework. 

## 5. Conclusions 

While there are a number of studies on biological robustness, the role of redundancy is still a theoretical debate in the phenomenon of biological robustness [55]. Our study provided a unique viewpoint of genetic robustness beyond duplication-based compensation and suggested the candidates of nonhomologous functional compensatory genes based on three features: (1) the existence of synthetic lethal interaction; (2) the ratio of shared common interacting partners; (3) the degree of coregulation. A candidate list was suggested for the future verification on the mechanisms of gene compensation (see the supplementary table). Several challenges still remain for understanding compensation mechanisms under different environmental and genetic variations. Functional compensation in SSL pairs might follow the four different mechanisms proposed by Kaelin: inclusion of direct surrogacy, subunits of an essential multiprotein complex, an essential linear pathway, and parallel pathways [56, 57]. Furthermore, the biological robustness of more organisms can be investigated through the concept of synthetic lethality, such as *Drosophila melanogaster*, *Caenorhabditis elegans,* and *Danio rerio* [57]. Future studies could focus on the mechanism for the compensation capabilities beyond the transcriptional level.

## Supplementary Material

The Supplementary table contains candidates of compensatory gene which identified by the features from this study.Most of gene pairs show high change of mRNA expression when a mutation or deletion of their SSL partner occurs. The citation numbers of references in the table are corresponding with the reference in the main text.
